# A placebo-controlled randomized HPV16 synthetic long-peptide vaccination study in women with high-grade cervical squamous intraepithelial lesions

**DOI:** 10.1007/s00262-012-1292-7

**Published:** 2012-06-09

**Authors:** Peggy J. de Vos van Steenwijk, Tamara H. Ramwadhdoebe, Margriet J. G. Löwik, Caroline E. van der Minne, Dorien M. A. Berends-van der Meer, Lorraine M. Fathers, A. Rob P. M. Valentijn, Jaap Oostendorp, Gert Jan Fleuren, Bart W. J. Hellebrekers, Marij J. P. Welters, Mariette I. van Poelgeest, Cornelis J. M. Melief, Gemma G. Kenter, Sjoerd H. van der Burg

**Affiliations:** 1 Department of Gynecology, Leiden University Medical Center, Leiden, The Netherlands; 2 Department of Clinical Oncology, Leiden University Medical Center, Building 1, K1-P, P.O. Box 9600, 2300 RC Leiden, The Netherlands; 3 Department of Clinical Pharmacology and Toxicology, Leiden University Medical Centre, Leiden, The Netherlands; 4 Department of Pathology, Leiden University Medical Centre, Leiden, The Netherlands; 5 Department of Immunohematology and Blood Transfusion, Leiden University Medical Centre, Leiden, The Netherlands; 6 Department of Obstetrics and Gynecology, Haga Teaching Hospital, The Hague, The Netherlands; 7 ISA Pharmaceuticals, Leiden, The Netherlands; 8 Present Address: CGOA, Amsterdam, The Netherlands

**Keywords:** HSIL, HPV16, Immunotherapy, Vaccination

## Abstract

The aim of this study was to investigate the capacity of an HPV16 E6/E7 synthetic overlapping long-peptide vaccine to stimulate the HPV16-specific T-cell response, to enhance the infiltration of HPV16-specific type 1 T cells into the lesions of patients with HPV16+ high-grade cervical squamous intraepithelial lesion (HSIL) and HPV clearance. This was a placebo-controlled randomized phase II study in patients with HPV16-positive HSIL. HPV16-specific T-cell responses were determined pre- and post-vaccination by ELISPOT, proliferation assay and cytokine assays in PBMC and HSIL-infiltrating lymphocytes, and delayed-type hypersensitivity skin tests. Motivational problems of this patient group to postpone treatment of their premalignant lesions affected the inclusion rates and caused the study to stop prematurely. Of the accrued patients, 4 received a placebo and 5 received 1–2 vaccinations. Side effects mainly were flu-like symptoms and injection site reactions. A strong HPV-specific IFNγ-associated T-cell response was detected by ELISPOT in all vaccinated patients. The outcome of the skin tests correlated well with the ELISPOT analysis. The cytokine profile associated with HPV16-specific proliferation varied from robust type 1 to dominant type 2 responses. No conclusions could be drawn on vaccine-enhanced T-cell infiltration of the lesion, and there was no HPV clearance at the time of LEEP excision. Thus, vaccination of HSIL patients results in increased HPV16-specific T-cell immunity. Further development of this type of treatment relies on the ability to motivate patients and in the reduction in the side effects.

**Electronic supplementary material:**

The online version of this article (doi:10.1007/s00262-012-1292-7) contains supplementary material, which is available to authorized users.

## Introduction

Persisting human papillomavirus (HPV) infections, in particular HPV type 16, are associated with the development of (pre)cancers of the anogenital tract. The risk of progression of squamous intraepithelial lesions (SIL) of the cervix is related to the severity of dysplasia [1, 2]. Small lesions are easily treated by loop electrosurgical excision procedure (LEEP), while larger lesions often require repeated surgery for recurrences [3]. Virus-specific, interferon-γ-producing CD4+ T cells and CD8+ cytotoxic T lymphocytes are essential components in controlling chronic viral infections [4, 5]. The majority of subjects who clear HPV16 display HPV16 E6-specific CD8+ cytotoxic T-lymphocyte (CTL) responses [6, 7], and relatively robust proliferative T-cell responses against early viral proteins E2, E6 and E7, characterized by CD4+ T cells that produce interferon-γ (IFNγ) and IL-5 [8–10]. Such IFNγ-associated T-cell responses are weak or absent in most patients with HSIL [7, 10–13].

Recently, two studies reported that therapeutic vaccination with an HPV16 E6/E7 protein or synthetic long-peptide vaccine (HPV16-SLP) resulted in the complete regression of HPV16-induced high-grade lesions of the vulva [14, 15]. Clinical success correlated with the induction of strong and broad HPV16-specific T-cell responses [14–16]. Non-responders had bigger lesions [15] and increased numbers of HPV-specific regulatory T cells [14, 16]. The aim of this study was to investigate the capacity of the HPV16-SLP vaccine to stimulate the HPV16-specific T-cell response in patients with HPV16+ high-grade lesions of the cervix.

## Materials and methods

### Patients and vaccination

This was a placebo-controlled randomized blinded phase II study aiming to include 34 patients, 17 in each arm. The aim of this study was to investigate the capacity of an HPV16 E6/E7 synthetic overlapping long-peptide vaccine to stimulate the HPV16-specific T-cell response, to enhance the infiltration of HPV16-specific type 1 T cells into the lesions of patients with HPV16+ high-grade cervical squamous intraepithelial lesion (HSIL) and HPV clearance.

**Fig. 1:**
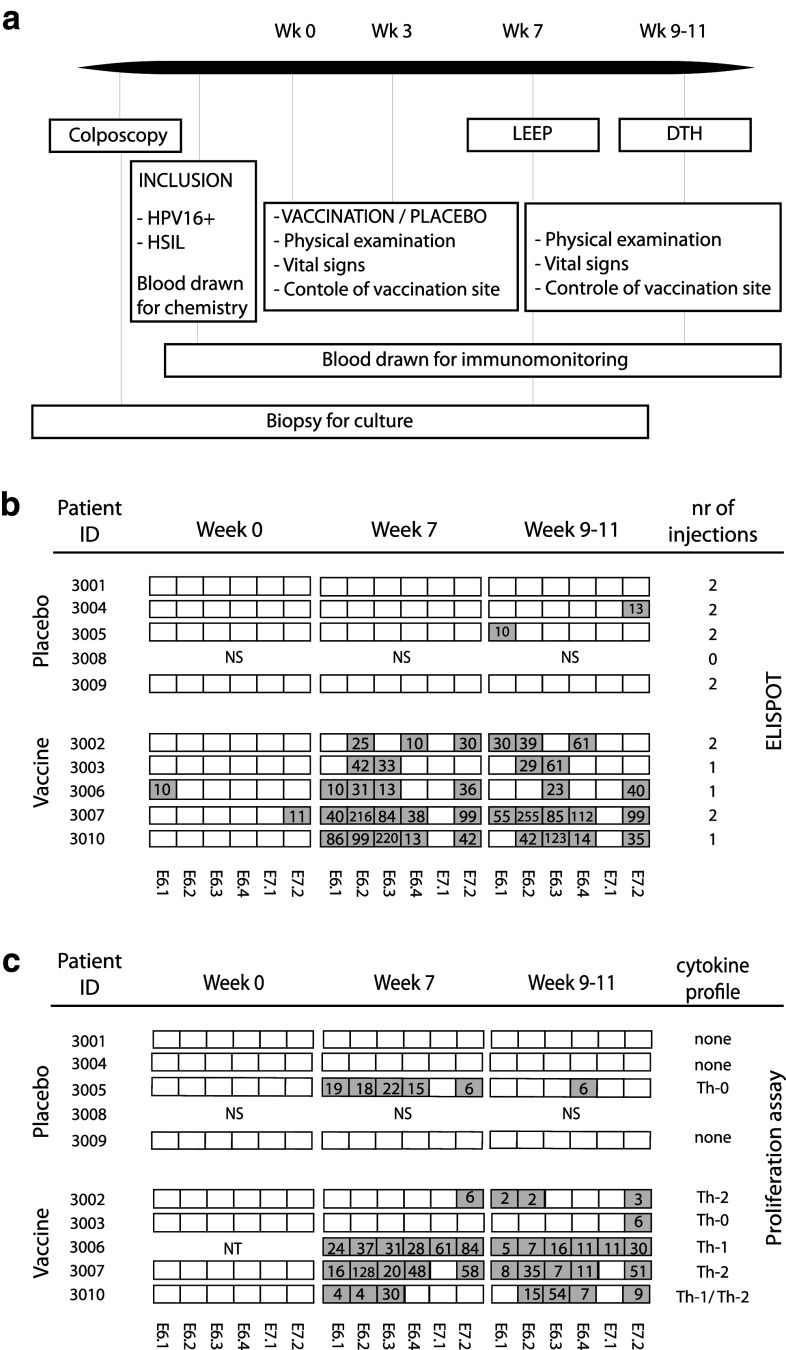
Schematic representation of the placebo-controlled randomized trial and summary of immunological results. **a** Patients were recruited at colposcopy visit. After informed consent, HPV testing was performed by PCR and an extra biopsy was taken for the culture of HSIL-infiltrating lymphocytes. Patients with histological proven HPV16+ HSIL then consented to the vaccination study at which time blood was drawn for chemistry and base-line immunomonitoring. Patients in arm 1 received the vaccine at a dose of 300 μg per peptide twice with a 3-week interval; patients in arm 2 received a placebo (PBS). Seven weeks after the first vaccination, a LEEP excision was performed at which time an extra biopsy of the HSIL was taken and blood drawn for immunomonitoring. DTH skin test was performed 2–4 weeks after surgery at which time blood was drawn to measure the effect of the LEEP excision on the systemic immune response. **b** Immunological results of all the patients using PBMC from three different time points. Week 0 (prevaccination), week 7 (post-vaccination) and week 9–11 (after LEEP excision). Systemic HPV16-specific T-cell reactivity against six peptide pools (4 E6 and 2 E7 peptide pools) was determined by IFNγ-ELISPOT. The boxes in gray show the number of HPV-specific IFNγ-producing T cells per 100,000 cells. **c** HPV specificity determined by the proliferation assay (LST). The gray boxes indicate the (stimulation index) SI of the HPV-specific proliferative responses. The culture at week 0 of patient 3006 was not tested due to technical problems. To the right, the overall cytokine profile based on the outcome of tested supernatants of the LST by cytokine bead array (CBA) is indicated. A Th-0 response indicates weak cytokine production inconclusive for a Th-1 or Th-2 response. Patient 3008 was randomized, but never showed up for vaccination; *NS,* not started

Patients with histologically proven HPV16+ HSIL were included after oral and written informed consent and randomized into two groups. Eligibility required pretreatment laboratory findings of leukocytes > 3 × 10^9^/L, lymphocytes > 1 × 10^9^/L, thrombocytes > 100 × 10^9^/L and hematocrit > 30 %, and no radiotherapy, chemotherapy or other potentially immunosuppressive therapy administered within 4 weeks prior to the immunotherapy. Patients consented to HPV testing and to having an extra biopsy taken for culture of HSIL-infiltrating lymphocytes at colposcopy (Fig. 1a). HPV typing was done on paraffin-embedded sections of biopsies or smears as published previously [17–19]. Patients received either the vaccine at a dose of 300 μg per peptide twice with a 3-week interval or a placebo, phosphate-buffered saline (PBS). Blood was drawn at week 0, 7 and 9–11. Both at screening and LEEP excision, an extra biopsy was taken for the culture of HSIL-infiltrating lymphocytes. A delayed-type hypersensitivity (DTH) skin test was performed 2–4 weeks after LEEP excision. The study was approved by the Dutch Central Committee on Human Research (CCMO, https://toetsingonline.ccmo.nl/ccmo_search.nsf/dossier number NL14015.000.06) and the Medical Ethical Committee of the Leiden University Medical Centre and the Haga Teaching Hospital. Monitoring for adverse events was performed as described previously [20], and adverse events were classified according to the Common Terminology Criteria for Adverse Events version 3.0 (CTCAE). The vaccine consisted of a mix of 13 overlapping 25–35 mer peptides representing the entire sequence of the E6 and E7 proteins of HPV16 (HPV16-SLP) dissolved in dimethylsulfoxide (DMSO) and admixed with 20 mM phosphate buffer (pH 7.5) and Montanide ISA-51. The vaccine was produced at the GMP facility of the Leiden University Medical Center (LUMC) [15, 16, 20, 21].

### T-cell assays, data acquisition, analysis and interpretation

The peripheral blood mononuclear cells (PBMCs) were tested for HPV16 specificity by a set of complementary T-cell immune monitoring assays including IFN-γ-ELISPOT, lymphocyte proliferation assay (LST) and cytokine bead array (CBA), using pools of 22 amino acid long peptides, overlapping by 12 amino acids. All tests have previously been described, and positive responses have been defined [22]. For all T-cell assays, a vaccine-induced response was defined as at least a threefold increase in the response after vaccination when compared to the results before vaccination. A semi-quantitative analysis of local changes in immune infiltrate was done on hematoxylin–eosine-stained sections before and after vaccination. HSIL-infiltrating lymphocytes were isolated, cultured and tested for HPV16-specific proliferation and cytokine production as described previously [16].

The T-cell assays were performed in the laboratory of the Department of Clinical Oncology (LUMC, Leiden) that operates under research conditions, following standard operating procedure (SOPs) and using trained staff. This laboratory has participated in all proficiency panels of the CIMT Immunoguiding Program (http://www.cimt.eu/workgroups/cip/), as well as in IFNγ ELISPOT panels of the Cancer Immunotherapy Consortium, which aim is to harmonize the reporting and assays used for T-cell monitoring [23–25].

### Delayed-type hypersensitivity skin tests

Delayed-type hypersensitivity reactions can be used as a sensitive and simple method for in vivo measurement of HPV-specific cellular immune responses and were used as previously described [26].

## Results

### Vaccinations

A total of 47 patients visiting the out-patient department of two hospitals in the Netherlands gave informed consent to screening for this study between June 2007 and December 2009. Due to the anxiety of patients with a HSIL to postpone the LEEP procedure, accrual proved an obstacle. Of the 27 eligible patients, only 10 consented and one patient (placebo group) never showed up for vaccination. Within the vaccine group, two patients (3006 and 3003) did not receive the second vaccination due to side effects and one (3010) due to a study stop (Fig. 1b).

### Safety and toxicity

Placebo patients did not display adverse reactions. As expected on the basis of our previous trials [15, 20, 21], all 5 patients in the vaccination group displayed adverse reactions not exceeding grade 2 according to CTCAE and included injection site reactions with itching, redness, swelling and pain. All patients experienced swellings of more than 8 cm which lasted for several days. Systemic reactions consisted of a headache (80 % of the patients), diarrhea, fatigue and/or dizziness (40 % of the patients) and nausea, chills, myalgia, rash, fever, urticaria, edema of the limbs or an allergic reaction needing antihistamines (20 % of the patients). Two patients (3002 and 3007) experienced stronger side effects after the second vaccination. In 4 cases, there was a renewed reaction to the vaccine 5–14 days after vaccination consisting of increased injection site reactions with or without systemic reactions. The skin test caused mainly itching at the site of the test.

### Spontaneous and vaccine-induced HPV16 E6- and E7-specific T-cell immunity

**Fig. 2:**
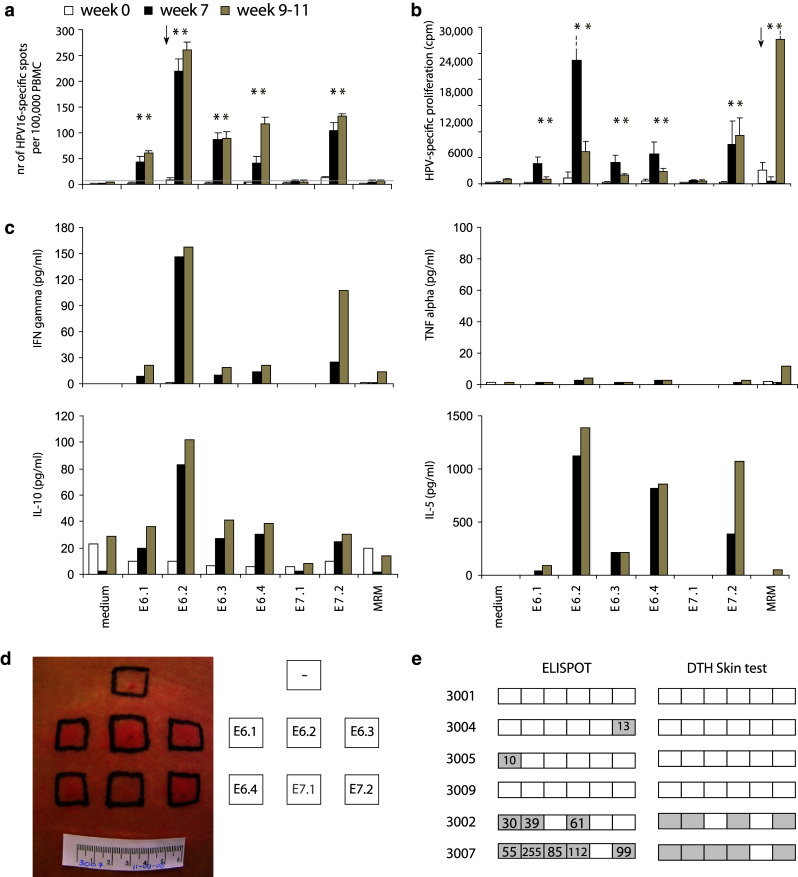
Example of the results from immunomonitoring. The results of patient 3007 who received two vaccinations and of whom blood was tested at week 0 (prevaccination; white), at week 7 (post-vaccination; black) and at week 9–11 (after LEEP excision; gray). The *arrows* indicate a preexisting response and the *stars* indicate a positive reaction during the course of the study. **a** Results of the IFNγ-ELISPOT assay. **b** Results of the proliferation assay showed no preexcising HPV-specific reactivity. **c** Cytokine bead array (CBA) was used to test the HPV16-specific production of the indicated cytokines measured in the culture supernatants of the proliferation assay. **d** DTH results showing clear redness and swelling of sites injected with E6.1, E6.2, E6.3, E6.4 and E7.2. **e** Overview of the IFNγ-ELISPOT results compared to the DTH skin test results

Systemic HPV16-specific T-cell reactivity from all three time points was simultaneously determined by IFNγ-ELISPOT (Figs. 1b and 2a, Online resource 1). Only two of the nine patients (3006 and 3007) showed a weak preexisting HPV16-specific immunity, one against E6 and one against E7 (10 and 11 spots per 100,000 PBMC). All vaccinated patients showed strong responses to 2–5 of the peptide pools (5/5 patients against E6 peptide pools and 4/5 against E7.2) 7 weeks after first vaccination, with reactivity up to 255 HPV-specific IFNγ-producing cells per 100,000 cells (Fig. 1b). Of the patients receiving placebo, 2 subjects (3004 and 3005) showed a weak IFNγ-associated HPV-specific response to one peptide pool (13 and 10 spots per 100,000 cells, against E7.2 and E6.1, respectively).

None of the patients tested displayed an HPV16-specific proliferative response at the start of the study. Three of the four patients receiving a placebo (3001, 3004 and 3009) remained unresponsive to HPV16 E6 and E7 throughout the duration of the study (Fig. 1c, Online resource 2). One patient (3005), received a placebo, yet developed a broad proliferative response after colposcopy with biopsy (week 7), which subsided after LEEP excision (week 9–11). All 5 vaccinated patients developed an HPV16-specific proliferative response after vaccination. Patients 3006 and 3007 developed the broadest responses to 5–6 peptide pools, and the other three patients responded to 1–4 pools (4/5 against E6 peptide pools and 5/5 against E7 peptide pools) at week 9–11. (Figure 1b and 2b, Online resource 2).

The supernatant of the proliferation assays was tested for the presence of HPV16-specific produced cytokines IFNγ, TNFα, IL-10, IL-5, IL-4 and IL-2. Before vaccination, no HPV16-specific cytokine production was found (Online resource 3 and Fig. 2c). At the time of LEEP treatment, HPV16-specific IFNγ production—ranging between 146 and 1582 pg/mL—was found in 3 of the five vaccinated patients (3006, 3007 and 3010). Only in one patient (3006) did we find a robust T-helper type 1 response (Online resource 3). Two patients displayed a Th-2 response (3002 and 3007) with the production of IL-5 and IL-10. One patient (3003) had a weak polarization (Th-0) with very low amounts of IL-5 production, and one patient (3010) had a weak mixed Th-1 and Th-2 response producing little amounts of IFNγ, TNFα, IL-5 and IL-10 (Online resource 3). Patient 3005 who was not vaccinated had a Th-0 response with very low amounts of IFNγ against one pool and IL-5 against another, despite a broad proliferative response. This is typical for HPV16-specific immunity in patients invasively treated for a persistent or recurrent lesion [13]. IL-4 or IL-2 is most likely consumed by T cells during the culture.

### Systemic immunity to recall antigens

In order to test the general immune status of the patients, the capacity of their T cells to proliferate and produce cytokines when stimulated with a mix of recall antigens (MRM) was tested. All patients, except 3002, displayed a proliferative response to MRM at all time points. MRM-specific IFNγ production was detected in the culture supernatants of patients 3001, 3003, 3005, 3006 and 3009 and in patient 3010 by IFNγ-ELISPOT. Patient 3004 failed to produce MRM-specific cytokines. Patient 3002 and 3007 produced IL-10 (33 pg/mL) and IL-5 (53 pg/mL), respectively.

### Local changes in HSIL biopsies

All patients were diagnosed with HSIL before vaccination. One patient (3001) in the placebo group had two biopsies (punch and LEEP) taken before the trial because of a discrepancy between the PAP smear (Pap4) and the first biopsy (no dysplasia, the second showed a CIN2). In the LEEP specimen after vaccination, no dysplasia was found. In none of the other patients was a change in the histological and viral disease status found at the time of LEEP. A semi-quantitative analysis of local changes in immune infiltrate on hematoxylin–eosine-stained sections revealed a change from a scattered pattern to a dense immune infiltrate in 3 (3002, 3003, 3010) of the 5 vaccinated patients. A similar change was observed in one (3004) of the three patients in the placebo group.

We received biopsies from all patients before vaccination for T-cell culture. After vaccination, we received biopsies from 3 placebo-treated patients and 3 vaccinated patients. In three of the 9 biopsies taken at the start of the study, enough T cells could be isolated to test for the presence of HPV16-specific T cells. Only in the culture of patient 3010 was a proliferative response detected against monocytes pulsed with the combined peptide pools E6.1 and 2 as well as against monocytes pulsed with protein. After vaccination, we received 6 biopsies for culture (3 from vaccinated patients and 3 from placebo-treated patients). Only two cultures, both from vaccinated patients (patient 3006 and 3003) had enough T cells to be tested. Neither showed evidence of HPV16 specificity. We did not receive a biopsy after vaccination from patient 3010 (who tested positive prevaccination).

### Responses to an HPV16 peptide-based skin test

Skin tests, based on DTH reactions against HPV16 peptides, can be used for in vivo measurement of HPV-specific cellular immunity [26]. Patients receiving placebo showed no skin reactions. Patients 3003, 3006 and 3010 who stopped after one vaccination did not receive the skin test. Patients 3002 and 3007 showed strong DTH reactivity after two vaccinations matching the results of the IFNγ-ELISPOT assay (Fig. 2d, e).

## Discussion

Therapeutic HPV vaccination is a promising strategy for HPV-induced precancerous lesions and cancer as shown for patients with high-grade VIN lesions by us and others [14, 15]. The aim of this study was to examine the systemic and local HPV16-specific T-cell responses after HPV16-SLP vaccination in patients with HPV16-induced HSIL. We were able to identify enough patients within 18 months, yet we experienced problems in accrual, due to patient anxiety at having to postpone standard surgical treatment. The study was extended in time; however, the accrual stayed extremely low and it was decided to stop the study prematurely. These problems have been described before in other attempts to test potential vaccines in patients with HSIL [27, 28]. Overall, the inclusion rate in this study was 19 %. This was quite unexpected as the inclusion rate in our previous trials in which this vaccine was tested in patients with cancer or VIN was well over 60 % [15, 20]. In contrast to patients with VIN3—for whom treatment is mutilating, disfiguring and of which the effects are mostly transient as recurrences are high [15, 20, 29]—this is not the case for patients with HSIL as they have no symptoms of their lesion and can be treated relatively easily by surgery. The side effects including among others swellings of 8 cm of the injection site and flu-like symptoms were expected on the basis of our earlier trials [15, 20, 21]. However, though they did not bear much impact on the study in patients with VIN3, it did cause a high drop out of patients in this trial. This clearly shows that strong disparities in the side effects and benefit between the standard of care and new therapies may outweigh the potential benefits of newly tested therapeutic modalities and affect clinical testing.

This was the first placebo-controlled trial with this HPV16-SLP vaccine. Although the numbers were small, it allowed us to show that the standard care, which includes a biopsy, can induce a broad and strong HPV16-specific response. However, this response was neither associated with the production of IFNγ nor with a positive skin test. In contrast to the placebo group, all vaccinated subjects displayed a strong vaccine-induced IFNγ-associated T-cell response as measured by ex vivo IFNγ-ELISPOT. This placebo-controlled trial thus sustains our notion to use the IFNγ-ELISPOT assay to determine vaccine-induced HPV16-specific T-cell reactions. The skin test assay may be an alternative as the pattern of skin reactions found in the 2 vaccinated and 4 placebo-treated patients tested matched well the results of the IFNγ-ELISPOT assay, confirming our previous observations that they have quite similar detection rates [26]. Notably, IFNγ-ELISPOT reactivity correlated with clinical responsiveness in our previous study in patients with vulvar lesions [15]. Only 1 vaccinated patient (3006) showed a robust Th-1 profile at week 7 after receiving only one vaccination.

An earlier randomized blinded placebo-controlled study with E6 and E7 protein in ISCOMATRIX in HSIL patients reported stronger HPV16-specific T-cell responses in immunized subjects than in placebo recipients. No clinical effects were observed [27]. In addition, a recent report on the use of an encapsulated plasmid DNA vaccine revealed that about half of the patients mounted a transient HPV-specific CD8 T-cell response [30]. Furthermore, HSIL patients vaccinated with a MVA viral vector expressing HPV16 E6 and E7 as well as IL-2 displayed some clinical efficacy at 6 months but the correlation with vaccine-induced T-cell reactivity was not assessed [28].

Overall, our placebo-controlled study shows that the HPV16-SLP vaccine is capable of increasing the numbers of circulating IFNγ-producing HPV16-specific T cells in patients with HSIL. These responses can be reliably detected using a DTH skin test. Importantly, motivational problems and the local and systemic side effects of the HPV16-SLP vaccine in HSIL patients must be taken into account when considering further studies in patients with premalignant lesions for whom an effective treatment is available. Future efforts should be focused on the development of a well-tolerated formulation, capable of inducing strong immune responses in patients with premalignant HPV-induced disease.

### Electronic supplementary material

Below is the link to the electronic supplementary material.

Supplementary material 1 (PDF 217 kb)

## References

[1] Smith JS, Lindsay L, Hoots B, Keys J, Franceschi S, Winer R, Clifford GM (2007). Human papillomavirus type distribution in invasive cervical cancer and high-grade cervical lesions: a meta-analysis update. Int J Cancer.

[2] Schiffman M, Kjaer SK (2003). Chapter 2: Natural history of anogenital human papillomavirus infection and neoplasia. J Natl Cancer Inst Monogr.

[3] Murdoch JB, Morgan PR, Lopes A, Monaghan JM (1992). Histological incomplete excision of CIN after large loop excision of the transformation zone (LLETZ) merits careful follow up, not retreatment. Br J Obstet Gynaecol.

[4] Matloubian M, Concepcion RJ, Ahmed R (1994). CD4+ T cells are required to sustain CD8+ cytotoxic T-cell responses during chronic viral infection. J Virol.

[5] Zajac AJ, Murali-Krishna K, Blattman JN, Ahmed R (1998). Therapeutic vaccination against chronic viral infection: the importance of cooperation between CD4+ and CD8+ T cells. Curr Opin Immunol.

[6] Nakagawa M, Stites DP, Farhat S, Sisler JR, Moss B, Kong F, Moscicki AB, Palefsky JM (1997). Cytotoxic T lymphocyte responses to E6 and E7 proteins of human papillomavirus type 16: relationship to cervical intraepithelial neoplasia. J Infect Dis.

[7] Nakagawa M, Stites DP, Patel S, Farhat S, Scott M, Hills NK, Palefsky JM, Moscicki AB (2000). Persistence of human papillomavirus type 16 infection is associated with lack of cytotoxic T lymphocyte response to the E6 antigens. J Infect Dis.

[8] de Jong A, van der Burg SH, Kwappenberg KM, van der Hulst JM, Franken KL, Geluk A, van Meijgaarden KE, Drijfhout JW, Kenter G, Vermeij P, Melief CJ, Offringa R (2002). Frequent detection of human papillomavirus 16 E2-specific T-helper immunity in healthy subjects. Cancer Res.

[9] Welters MJ, de Jong A, van den Eeden SJ, van der Hulst JM, Kwappenberg KM, Hassane S, Franken KL, Drijfhout JW, Fleuren GJ, Kenter G, Melief CJ, Offringa R, van der Burg SH (2003). Frequent display of human papillomavirus type 16 E6-specific memory t-Helper cells in the healthy population as witness of previous viral encounter. Cancer Res.

[10] de Jong A, van Poelgeest MI, van der Hulst JM, Drijfhout JW, Fleuren GJ, Melief CJ, Kenter G, Offringa R, van der Burg SH (2004). Human papillomavirus type 16-positive cervical cancer is associated with impaired CD4+ T-cell immunity against early antigens E2 and E6. Cancer Res.

[11] Bontkes HJ, de Gruijl TD, Bijl A, Verheijen RH, Meijer CJ, Scheper RJ, Stern PL, Burns JE, Maitland NJ, Walboomers JM (1999). Human papillomavirus type 16 E2-specific T-helper lymphocyte responses in patients with cervical intraepithelial neoplasia. J Gen Virol.

[12] Nimako M, Fiander AN, Wilkinson GW, Borysiewicz LK, Man S (1997). Human papillomavirus-specific cytotoxic T lymphocytes in patients with cervical intraepithelial neoplasia grade III. Cancer Res.

[13] de Vos van Steenwijk PJ, Piersma SJ, Welters MJ, van der Hulst JM, Fleuren G, Hellebrekers BW, Kenter GG, van der Burg SH (2008). Surgery followed by persistence of high-grade squamous intraepithelial lesions is associated with the induction of a dysfunctional HPV16-specific T-cell response. Clin Cancer Res.

[14] Daayana S, Elkord E, Winters U, Pawlita M, Roden R, Stern PL, Kitchener HC (2010). Phase II trial of imiquimod and HPV therapeutic vaccination in patients with vulval intraepithelial neoplasia. Br J Cancer.

[15] Kenter GG, Welters MJ, Valentijn AR, Lowik MJ, Berends-van der Meer DM, Vloon AP, Essahsah F, Fathers LM, Offringa R, Drijfhout JW, Wafelman AR, Oostendorp J, Fleuren GJ, van der Burg SH, Melief CJ (2009). Vaccination against HPV-16 oncoproteins for vulvar intraepithelial neoplasia. N Engl J Med.

[16] Welters MJ, Kenter GG, de Vos van Steenwijk PJ, Lowik MJ, Berends-van der Meer DM, Essahsah F, Stynenbosch LF, Vloon AP, Ramwadhdoebe TH, Piersma SJ, van der Hulst JM, Valentijn AR, Fathers LM, Drijfhout JW, Franken KL, Oostendorp J, Fleuren GJ, Melief CJ, van der Burg SH (2010). Success or failure of vaccination for HPV16-positive vulvar lesions correlates with kinetics and phenotype of induced T-cell responses. Proc Natl Acad Sci USA.

[17] Tieben LM, ter Schegget J, Minnaar RP, Bouwes Bavinck JN, Berkhout RJ, Vermeer BJ, Jebbink MF, Smits HL (1993). Detection of cutaneous and genital HPV types in clinical samples by PCR using consensus primers. J Virol Methods.

[18] Roda Husman AM, Walboomers JM, Van Den Brule AJ, Meijer CJ, Snijders PJ (1995). The use of general primers GP5 and GP6 elongated at their 3′ ends with adjacent highly conserved sequences improves human papillomavirus detection by PCR. J Gen Virol.

[19] Van Den Brule AJ, Pol R, Fransen-Daalmeijer N, Schouls LM, Meijer CJ, Snijders PJ (2002). GP5+/6+ PCR followed by reverse line blot analysis enables rapid and high-throughput identification of human papillomavirus genotypes. J Clin Microbiol.

[20] Kenter GG, Welters MJ, Valentijn AR, Lowik MJ, Berends-van der Meer DM, Vloon AP, Drijfhout JW, Wafelman AR, Oostendorp J, Fleuren GJ, Offringa R, van der Burg SH, Melief CJ (2008). Phase I immunotherapeutic trial with long peptides spanning the E6 and E7 sequences of high-risk human papillomavirus 16 in end-stage cervical cancer patients shows low toxicity and robust immunogenicity. Clin Cancer Res.

[21] Welters MJ, Kenter GG, Piersma SJ, Vloon AP, Lowik MJ, Berends-van der Meer DM, Drijfhout JW, Valentijn AR, Wafelman AR, Oostendorp J, Fleuren GJ, Offringa R, Melief CJ, van der Burg SH (2008). Induction of tumor-specific CD4+ and CD8+ T-cell immunity in cervical cancer patients by a human papillomavirus type 16 E6 and E7 long peptides vaccine. Clin Cancer Res.

[22] Speetjens FM, Kuppen PJ, Welters MJ, Essahsah F, Voet van den Brink AM, Lantrua MG, Valentijn AR, Oostendorp J, Fathers LM, Nijman HW, Drijfhout JW, van de Velde CJ, Melief CJ, van der Burg SH (2009). Induction of p53-specific immunity by a p53 synthetic long peptide vaccine in patients treated for metastatic colorectal cancer. Clin Cancer Res.

[23] Britten CM, Gouttefangeas C, Welters MJ, Pawelec G, Koch S, Ottensmeier C, Mander A, Walter S, Paschen A, Muller-Berghaus J, Haas I, Mackensen A, Kollgaard T, Thor SP, Schmitt M, Giannopoulos K, Maier R, Veelken H, Bertinetti C, Konur A, Huber C, Stevanovic S, Wolfel T, van der Burg SH (2008). The CIMT-monitoring panel: a two-step approach to harmonize the enumeration of antigen-specific CD8+ T lymphocytes by structural and functional assays. Cancer Immunol Immunother.

[24] Moodie Z, Price L, Gouttefangeas C, Mander A, Janetzki S, Lower M, Welters MJ, Ottensmeier C, van der Burg SH, Britten CM (2010). Response definition criteria for ELISPOT assays revisited. Cancer Immunol Immunother.

[25] Britten CM, Janetzki S, van der Burg SH, Huber C, Kalos M, Levitsky HI, Maecker HT, Melief CJ, O’Donnell-Tormey J, Odunsi K, Old LJ, Pawelec G, Roep BO, Romero P, Hoos A, Davis MM (2011). Minimal information about T cell assays: the process of reaching the community of T cell immunologists in cancer and beyond. Cancer Immunol Immunother.

[26] van den Hende M, van Poelgeest MI, van der Hulst JM, de Jong J, Drijfhout JW, Fleuren GJ, Valentijn AR, Wafelman AR, Slappendel GM, Melief CJ, Offringa R, van der Burg SH, Kenter GG (2008). Skin reactions to human papillomavirus (HPV) 16 specific antigens intradermally injected in healthy subjects and patients with cervical neoplasia. Int J Cancer.

[27] Frazer IH, Quinn M, Nicklin JL, Tan J, Perrin LC, Ng P, O’Connor VM, White O, Wendt N, Martin J, Crowley JM, Edwards SJ, McKenzie AW, Mitchell SV, Maher DW, Pearse MJ, Basser RL (2004). Phase 1 study of HPV16-specific immunotherapy with E6E7 fusion protein and ISCOMATRIX adjuvant in women with cervical intraepithelial neoplasia. Vaccine.

[28] Brun JL, Dalstein V, Leveque J, Mathevet P, Raulic P, Baldauf JJ, Scholl S, Huynh B, Douvier S, Riethmuller D, Clavel C, Birembaut P, Calenda V, Baudin M, Bory JP (2011). Regression of high-grade cervical intraepithelial neoplasia with TG4001 targeted immunotherapy. Am J Obstet Gynecol.

[29] van Seters M, van Beuren M, de Craen AJ (2005). Is the assumed natural history of vulvar intraepithelial neoplasia III based on enough evidence? A systematic review of 3322 published patients. Gynecol Oncol.

[30] Matijevic M, Hedley ML, Urban RG, Chicz RM, Lajoie C, Luby TM (2011). Immunization with a poly (lactide co-glycolide) encapsulated plasmid DNA expressing antigenic regions of HPV 16 and 18 results in an increase in the precursor frequency of T cells that respond to epitopes from HPV 16, 18, 6 and 11. Cell Immunol.

