# Supplementary material for: A placebo-controlled randomized HPV16 synthetic long-peptide vaccination study in women with high-grade cervical squamous intraepithelial lesions
Source: Cancer Immunol Immunother. 2012 Jun 9;61(9):1485–92. doi: 10.1007/s00262-012-1292-7 (PMC3427705; doi:10.1007/s00262-012-1292-7)
Supplement: Supplementary file 1 — Supplementary material 1 (PDF 217 kb) [file 262_2012_1292_MOESM1_ESM.pdf]

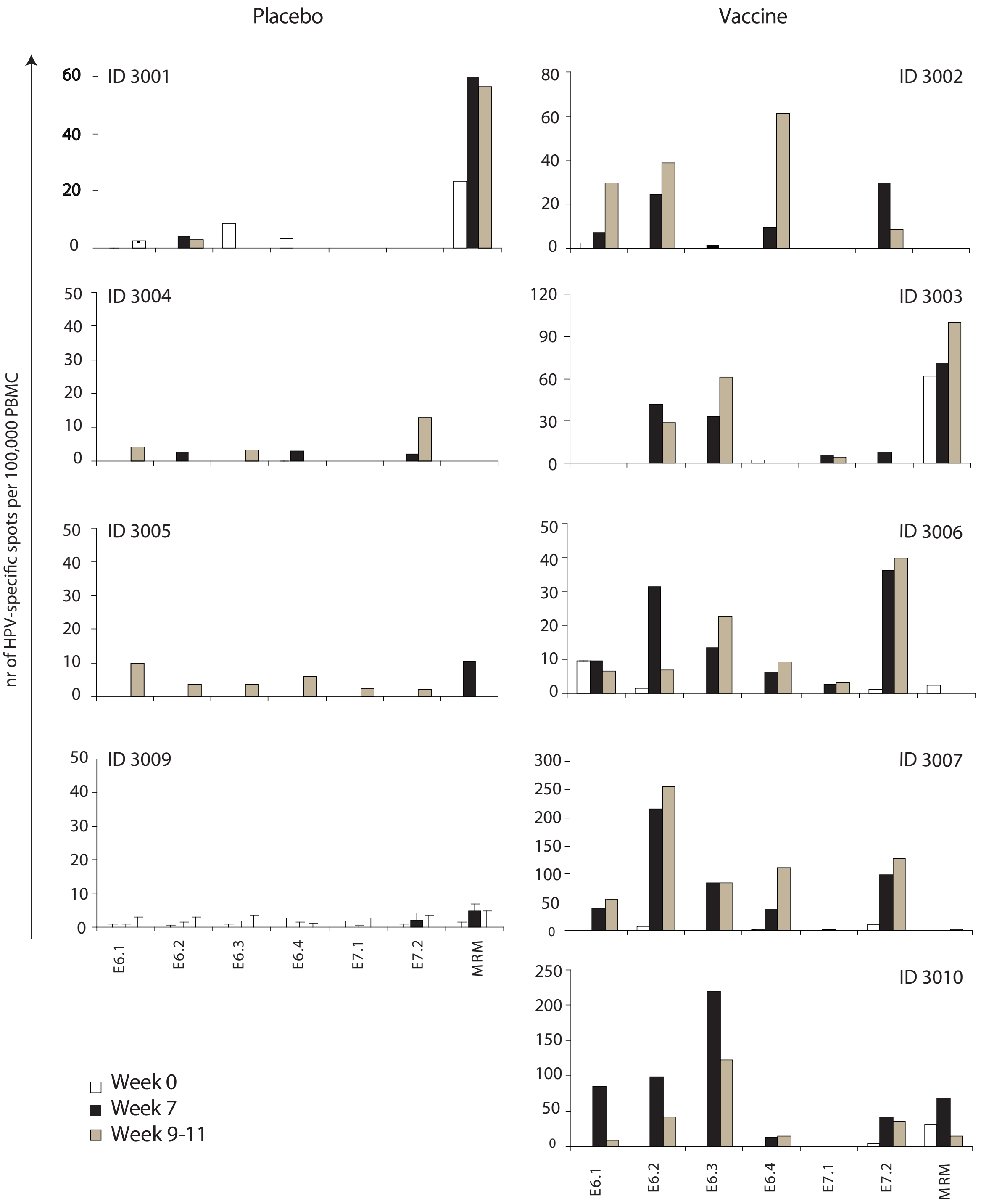

Online Resource 1. Immunomonitoring by IFNγ-ELISPOT. The patients who received a placebo are shown at the left and at the right are the patients who received the vaccine. The results, week 0 (pre-vaccination), week 7 (post-vaccination) and week 9-11 (after LEEP excision), are depicted in one graph per patient. The results are shown as number of HPV-specific spot forming T-cells per 100,000 PBMC.

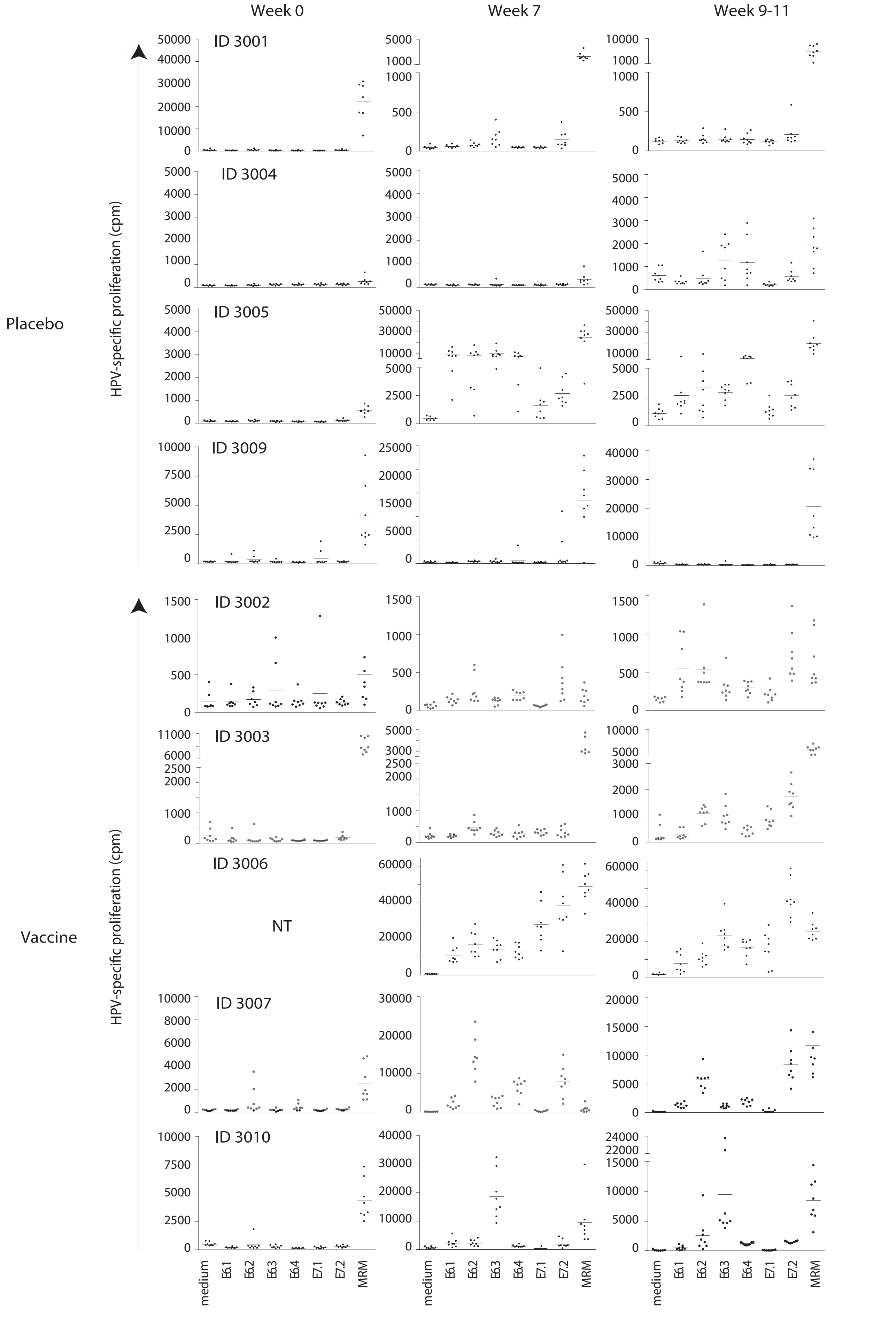

Online Resouce 2. Immunomonitoring by proliferation assay (LST). The top four patients received a placebo and the bottom five patients received the vaccine. The proliferative responses were measured after 7 days of culture by [3H]-thymidine incorporation for the last 24 hours and are displayed as count per minute (CPM). The results are shown for week 0 (pre-vaccination), week 7 (post-vaccination) and week 9-11 (after LEEP excision). The data of patient 3006 at week 0 were non evaluable due to a technical problem

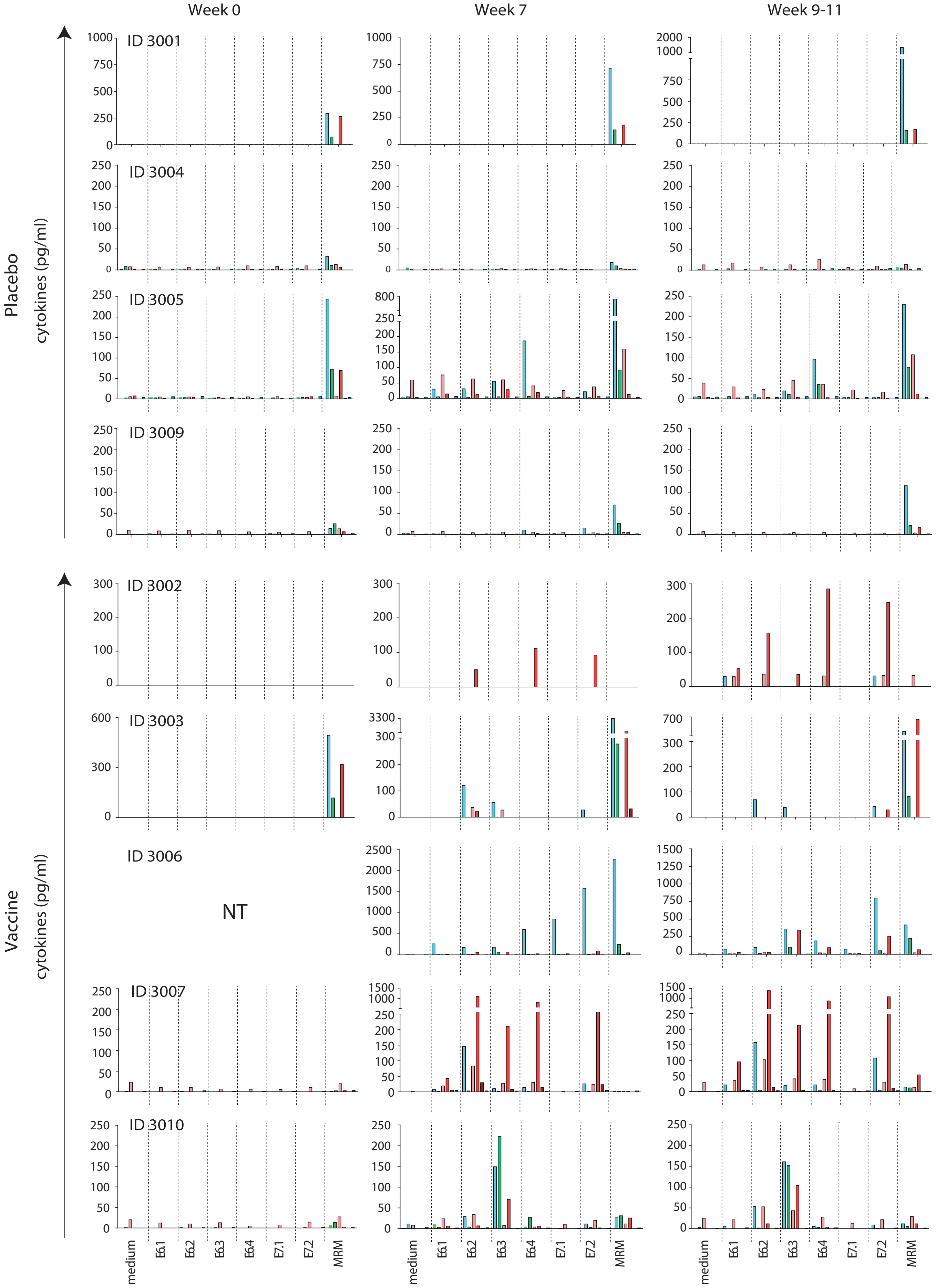

Online Resource 3. Immunomonitoring by cytokine bead array (CBA). Supernatants of the 7-day proliferative cultures (LST) were harvested at day 6 and subjected to CBA to measure the production of IFNγ (light blue), TNFα (dark green), IL-10 (pink), IL-5 (red), IL-4 (brown) IL-2 (dark blue). The results are shown for the three time points. The upper 4 patients received placebo the lower 5 received the vaccine. No results are available for patient 3006 at week 0.
